# Influencing factors of nonclinical pharmacists’ willingness to transform: a cross-sectional survey in Xinjiang, China

**DOI:** 10.1186/s12913-023-09667-2

**Published:** 2023-06-23

**Authors:** Tiantian Kong, Yuankai Huang, Xin Chen, Wenbing Yao

**Affiliations:** 1 Department of Pharmacy, Xinjiang Medical University, NO.393 Xinyi Road, Urumqi City, 830017 Xinjiang Province China; 2 School of International Pharmaceutical Business, China Pharmaceutical University, NO639 Lonmian Avenue, Jiangning District, Nanjing City, 211198 Jiangsu Province China; 3 Research Center of National Drug Policy & Ecosystem, China Pharmaceutical University, NO.639 Lonmian Avenue, Jiangning District, Nanjing City, 211198 Jiangsu Province China

**Keywords:** Clinical pharmacist, Pharmaceutical care, Willingness to transform, Influencing factors

## Abstract

**Background:**

There is a serious shortage of clinical pharmacists in Xinjiang, China. A six-month to one-year on-the-job training programme can rapidly transition nonclinical pharmacists into clinical pharmacists to resolve this issue. However, not all nonclinical pharmacists are willing to become clinical pharmacists, and many factors may influence their willingness. This study aims to assess the transformation intention of nonclinical hospital pharmacists and the contributing elements to make recommendations to accelerate the transformation of hospital pharmacists to clinical pharmacists.

**Methods:**

Cross-sectional survey was conducted in secondary and tertiary hospitals in Xinjiang. Taking 14 prefectures in Xinjiang as a cluster, 34 hospitals were randomly selected. By snowball sampling, the heads of pharmaceutical departments introduced non-clinical pharmacists to participate in an anonymous questionnaire survey, which included 41 questions about basic demographic information, cognition and attitudes towards pharmaceutical care, potential factors and willingness to transform, and it took an average of 10 min to complete. Using multifactor logistic regression, the contributing elements of transformation intention were analysed.

**Results:**

The survey was conducted from May to October 2022. 338 valid responses were obtained, with a response rate of 91.85% and a willingness to transform rate of 81.67%. There were significant differences in the willingness to transform among nonclinical pharmacists of different ages (*P* < 0.05), marital statuses (*P* < 0.05), years of employment (*P* < 0.05), and technical titles (*P* < 0.05). There were significant differences between the two groups in the following four aspects: whether the setting of human resources in the pharmaceutical department was reasonable (*P* < 0.05), the educational level of clinical pharmacists (*P* < 0.05), the higher salary level of clinical pharmacists (*P* < 0.05), and whether they had experience in pharmaceutical care (*P* < 0.05). There was a significant difference in the total score of the pharmaceutical care attitude scale (*P* < 0.05) between the willing and unwilling groups. The results of multivariate logistic regression analysis revealed that the experience of providing pharmaceutical care (*OR* = 4.601, 95% CI: 1.13–18.69, *P* < 0.05) and attitude towards pharmaceutical care (*OR* = 3.302, 95% CI: 1.19–9.19, *P* < 0.05) had a statistically significant influence on the transformation intention of nonclinical pharmacists.

**Conclusions:**

One-fifth of nonclinical pharmacists were unwilling to transition to clinical pharmacists. The attitude towards and experience of pharmaceutical care affected pharmacists’ transformation intention, so the suggestion is proposed to promote the transformation of nonclinical pharmacists into clinical pharmacists.

## Background

Pharmaceutical care (PC) is a pharmacist’s contribution to individual care with the goal of optimizing medication use and improving health outcomes [1]. Britain, the United States, and other countries have established relatively advanced PC systems. Comparatively, China's PC development is still in its early stage; many pharmacists still view drug supply as their primary responsibility, and the number of clinical pharmacists is insufficient [2]. As China's medical and health care reform continues to progress, advances in technology, such as intelligent and automated pharmacies, are becoming more commonplace in hospitals. This allows pharmacists to devote less time to drug supply and allocation and more time to "patient-centred" care while still actively promoting safe and effective medication administration.

Xinjiang is located in northwest China with a population of 25.85 million and an area of 1.6649 million square kilometres, accounting for one-sixth of China’s total area. Due to the constraints of the natural environment and geographical location, it is challenging to introduce skills in Xinjiang, and the talent needed is mainly trained by local colleges. For example, 70% of pharmaceutical graduates from Xinjiang Medical University work locally [3]. However, the universities in Xinjiang were not qualified to train clinical pharmacists until 2017, and the first batch of undergraduates graduated in July 2022 after completing five years of study. In accordance with *The Provisions on the Administration of Pharmaceutical Affairs in Medical Institutions*, secondary and tertiary hospitals are required to employ three and five clinical pharmacists, respectively [4], which is challenging in Xinjiang. China has designed a clinical pharmacist post-training programme to increase the number of clinical pharmacists and continually satisfy the growing demand for health services among the population. The specialist training cycle lasts one year, the general training cycle lasts six months, and the clinical pharmacist post-training certificate can be earned after successfully completing an examination [2]. Compared with other methods, such as full-time higher education for four to six years, the transformation of nonclinical pharmacists into clinical pharmacists through on-the-job training can not only alleviate the shortage of clinical pharmacists in hospitals as quickly as possible but also rationalize the distribution of hospital pharmacy staff. Currently, Xinjiang has 13 job training bases [5].

However, there are many factors that affect the transition of nonclinical pharmacists to clinical pharmacists. According to the knowledge-attitude/belief-practice (KABP/KAP) theory, knowledge is the basis for establishing a correct attitude and changing practice, while attitude is the driving force of practice change and knowledge and attitude are the necessary conditions for producing practice [6]. Therefore, empirical research based on the KAP theory and a comprehensive quantitative analysis of the factors that affect the transformation willingness of nonclinical pharmacists can provide evidence and verification for existing theoretical research [7–9]. In addition, due to the restrictions of the geographical environment, sparse population, and relatively underdeveloped economy in Xinjiang, research on pharmacist transformation remains unexplored.

This study investigated secondary and tertiary hospitals in Xinjiang, analysed the influencing factors with regard to subjective factors (such as cognition, attitude, and potential factors) and objective factors (such as age, professional title, and marriage) and proposed countermeasures to hasten the transformation of hospital pharmacists and promote the transformation of the pharmaceutical mode.

## Method

### Research framework

**Fig. 1:**
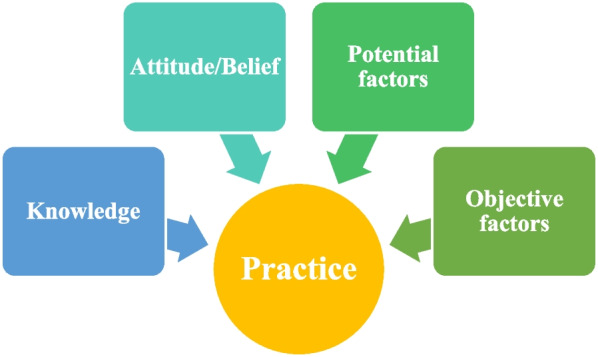
KAP model-based research approach for influencing factors

The design of this study is mainly based on the KAP theoretical model to explain how personal knowledge and attitudes affect practice change. In addition to knowledge and attitude, it covers aspects that may affect the transformation willingness of nonclinical pharmacists, such as demographic characteristics and other potentially subjective factors, including salary expectations, views on the allocation of human resources, perspectives on continuing education, and experience in PC, to ensure the completeness of the influencing factors. Therefore, the questionnaire survey of influencing factors in this study included four aspects: objective factors, cognition of clinical PC, attitude towards clinical PC, and other potential factors, as illustrated in Fig. 1.

### Questionnaire design and verification

The questionnaire design was based on successful cases of PC research [10, 11] and was combined with the actual situation in Xinjiang. After iterative review by three experts from the Clinical Pharmacy Professional Committee and two preinvestigations, items that may be potential influencing factors were added, and the problem expression was constantly improved. The questionnaire consisted of four parts. The first part collected the basic information of the subjects, including seven questions such as hospital grade, sex, age, professional title, and educational background. The second part collected potential factors, including nine questions such as views on the allocation of human resources, continuing education, salary expectations, and PC experience. The third section consisted of 12 questions pertaining to the concepts and guidelines of PC, with "Yes" and "No" as acceptable responses. The fourth part assessed attitudes towards PC using the Pharmacists’ Attitude Scale (PCAS) [11, 12] with 13 items and a 5-level Likert rating scale. The attitude dimension ranged from 1 “very much disagree” to 5 “very much agree,” with a total score of 13–65. In the questionnaire, the willingness of nonclinical pharmacists to become clinical pharmacists was measured by the question “whether you want to be a clinical pharmacist or not.” There were two response options: “Yes” and “No.”

Before the formal investigation, Cronbach’s alpha coefficient was 0.793, which was used to test the internal consistency of the Pharmacists’ Attitude Scale, indicating that the reliability was good. The validity of the scale was analysed, and the KMO test coefficient was 0.934, Bartlett’s spherical test *P* < 0.001, and the relative fit index (CFI) was 0.922, indicating that the validity of the scale was good.

### Sample criteria and sampling

The inclusion criteria of the study were as follows: (1) pharmacists who did not work in a clinical pharmacy in a hospital and had been employed for more than one year and (2) pharmacists who volunteered to participate in the study and signed the informed consent document. The exclusion criteria were part-time pharmacists and trainee pharmacists.

Cluster random sampling and snowball sampling were adopted, and the detailed strategies were as follows. From May to October 2022, the sample of the study came from all prefectures and cities in Xinjiang. Taking each prefecture and city in Xinjiang as a whole group, at least one local secondary hospital and at least one local tertiary hospital were randomly selected in each group. After communicating with the head of the hospital pharmaceutical department, the snowball method was used to recruit pharmacists to participate in the research with their informed consent. As the number of pharmacists in tertiary hospitals was more than in secondary hospitals, at least three pharmacists were selected in tertiary hospitals, and at least one pharmacist was selected in secondary hospitals. Of course, more voluntary participants were better. Finally, a total of 338 samples from various prefectures and cities in Xinjiang were collected, including 242 in tertiary hospitals and 96 in secondary hospitals.

### Data collection

Data were collected by online survey. The head of the hospital’s pharmaceutical department was approached for permission to post the Questionnaire Star online on WeChat (Wechat is a mobile messaging app that allows single or multiple people to send voice, picture, video, text and links over mobile networks), and pharmacists were invited to read the electronic version of the informed consent and voluntarily participate in the questionnaire. The collected electronic questionnaire can identify whether participant information meets the requirements in the background. Finally, 368 questionnaires were collected, and 30 invalid questionnaires were excluded. Of these, 5 respondents took less than 150 s to answer, 9 questionnaires had the same answers, and 16 questionnaires had logical errors. With a 91.85% effective response rate, 338 valid questionnaires were obtained.

To ensure the quality of the questionnaire, researchers should be given unified training before the start of pre-research and formal research. Upgrading Questionnaire Star from the basic version to the enterprise edition opened up many new functions for quality control, such as setting multiple-choice topics to random options and avoiding the information bias of selecting only the first few items of multiple-choice questions. When the reverse question was set, the system automatically identified a questionnaire that was not thoroughly read and randomly answered according to the reverse question and immediately marked it invalid. The minimum answer time was set, and after extensive pre-research, the system automatically identified an invalid questionnaire if the answer time was lower than a certain period. The questionnaire response area was limited to “Xinjiang.” Finally, valid questionnaires were screened manually to delete questionnaires that did not meet the requirements to ensure the quality of the survey.

### Data analysis

The data were derived from Questionnaire Star and analysed using SPSS 25.0. Statistical analysis methods included descriptive statistical analysis, *t* test, chi-square test, rank sum test, univariate regression analysis, multivariate logistic regression analysis, reliability, and validity test.

The following statistical method was used for comparison between the unintentional and intentional groups. (1) The quantitative data that satisfied a normal distribution were described by $\overline{x } \pm s$. Two independent-sample *t* tests were used for comparisons between the two groups. The test statistic was *t.* The significance level was defined as *P* < 0.05. (2) The quantitative data that did not satisfy the normal distribution were described by M (P_25_ P_75_). The group rank sum test was used to compare the two groups. The test statistic was *z*. The significance level was defined as *P* < 0.05. (3) The qualitative data were described by n (%), and the chi-square test was used for comparisons between the groups. The test statistic was ${\upchi }^{2}$ The significance level was defined as *P* < 0.05. (4) The ordered data were described by n (%), and the group rank sum test was used to compare the two groups. The test statistic was *z*. The significance level was defined as *P* < 0.05.

For the analysis of the influencing factors, logistic regression analysis was conducted with willingness as the outcome variable. Advanced univariate logistic regression was performed, and the variables with *P* < 0.05 were included in multivariate logistic regression. The significance level was defined as *P* < 0.05.

## Results

### Demographic characteristics and willingness to transform

Valid questionnaires were obtained from 338 nonclinical pharmacists from 34 hospitals in 14 prefectures in Xinjiang, with tertiary hospitals accounting for 71.6%, secondary hospitals accounting for 28.41%, men accounting for 31.95%, women accounting for 68.05%, unmarried respondents accounting for 23.96%, and married respondents accounting for 71.6%. The distribution of academic qualifications was as follows: master’s degree 8.58%, bachelor’s degree 72.78%, and below bachelor’s degree 18.64%. Professional titles were distributed as follows: 47.34% junior titles, 41.72% intermediate titles, 7.69% deputy senior titles, and 3.25% senior titles (Table 1).

**Table 1:** Comparison of nonclinical pharmacists’ willingness to transform based on demographic characteristics

**Factors**	**Unwilling group (*n* = 62)**	**Willing group (*n *= 276)**	**Total (*n* = 338)**	$\chi^2/\mathrm z$	***P***
**Grade of the hospital, n (%)**					
Tertiary	48 (77.42)	194 (70.29)	242 (71.59)	1.020	0.308
Secondary	14 (22.58)	82 (29.71)	96 (28.41)		
**Gender, n (%)**					
Male	23 (37.1)	85(30.8)	108 (31.95)	0.924	0.336
Female	39 (62.9)	191 (69.2)	230 (68.05)		
**Age, M (P**_**25**_**, P**_**75**_**)**	39 (33,49)	34 (28,40)	35 (29,41)	3.514	< 0.001^*^
**Marital status, n (%)**					
Unmarried	8 (12.9)	73 (26.45)	81 (23.96)	5.748	0.049^*^
Married	52 (83.87)	190 (68.84)	242 (71.6)		
Other status (divorced or widowed)	2 (3.23)	13 (4.71)	15 (4.44)		
**Working years, M (P**_**25**_**, P**_**75**_**)**	13 (8,23)	10 (3,15)	10 (4,16)	2.998	0.003^*^
**Education, n (%)**					
Below bachelor’s degree	10 (16.13)	53 (19.2)	63 (18.64)	0.616	0.538
Bachelor’s degree	46 (74.19)	200 (72.46)	246 (72.78)		
Master’s degree	6 (9.68)	23 (8.33)	29 (8.58)		
**Professional title, n (%)**					
Junior	20 (32.26)	140 (50.72)	160 (47.34)	3.145	0.002^*^
Intermediate	29 (46.77)	112 (40.58)	141 (41.72)		
Deputy senior	8 (12.9)	18 (6.52)	26 (7.69)		
Senior	5 (8.06)	6 (2.17)	11 (3.25)		

Remarks: * *Indicates P* < 0.05, and the difference is statistically significant

As illustrated in Table 1, 276 pharmacists were willing to transform, accounting for 81.67%. There were significant differences in the willingness to transform among nonclinical pharmacists with different ages (*P* < 0.05), marital statuses (*P* < 0.05), years of employment (*P* < 0.05), and technical titles (*P* < 0.05). There was no significant difference in the willingness to transform among nonclinical pharmacists with different hospital grades (*P* > 0.05), genders (*P* > 0.05), and academic qualifications (*P* > 0.05).

### Potential factors and willingness to transform

To preliminarily understand whether the potential factors affected the transition intention, the differences between the willing group and the unwilling group were compared. The results indicate that there are significant differences between whether the setting of human resources in the pharmaceutical department is reasonable (*P* < 0.05), the educational level of clinical pharmacists who need to be introduced (*P* < 0.05), the higher salary level of clinical pharmacists (*P* < 0.05) and whether they have experience in PC (*P* < 0.05) (Table 2).

**Table 2:** Comparison of nonclinical pharmacists’ willingness to transform on potential factors

**Factors**	**Unwilling group (*n* = 62)**	**Willing group (*n* = 276)**	**Total (*n *= 338)**	$\chi^2/\mathrm z$	***P***
If the allocation of human resources in the pharmaceutical department is reasonable. n (%)					
Yes	34 (54.84)	188 (68.12)	222 (65.68)	3.959	0.047^*^
No	28 (45.16)	88 (31.88)	116 (34.32)		
If the equipment of clinical pharmacists can meet the needs of guiding the use of drugs. n (%)					
Yes	39 (62.9)	172 (62.32)	211 (62.43)	0.007	0.932
No	23 (37.1)	104 (37.68)	127 (37.57)		
If the pharmaceutical department needs to introduce clinical pharmacists. n (%)					
Yes	55 (88.71)	246 (89.13)	301 (89.05)	0.009	0.924
No	7 (11.29)	30 (10.87)	37 (10.95)		
What kind of clinical pharmacist to introduce? n (%)					
Doctor’s degree	19 (30.65)	29 (10.51)	48 (14.20)	3.072	0.002^*^
Master’s degree	19 (30.65)	114 (41.3)	133 (39.35)		
Bachelor’s degree	17 (27.42)	89 (32.25)	106 (31.36)		
Below bachelor’s degree	0 (0.00)	14 (5.07)	14 (4.14)		
If the professional knowledge of clinical pharmacists can meet the needs of their positions. n (%)					
Yes	44 (70.97)	198 (71.74)	242 (71.6)	0.015	0.903
No	18 (29.03)	78 (28.26)	96 (28.4)		
If the existing clinical pharmacists must pursue further studies in academic education. n (%)					
Yes	56 (90.32)	253 (91.67)	309 (91.42)	0.117	0.733
No	6 (9.68)	23 (8.33)	29 (8.58)		
If the existing clinical pharmacists need continuing education and training. n (%)					
Yes	61 (98.39)	273 (98.91)	334 (98.82)	-	0.557
No	1 (1.61)	3 (1.09)	4 (1.18)		
If the salaries of clinical pharmacists are higher than yours. n (%)					
Yes	20 (32.26)	137 (49.64)	157 (46.45)	6.644	0.036^*^
No	13 (20.97)	36 (13.04)	49 (14.5)		
Don’t know	29 (46.77)	103 (37.32)	132 (39.05)		
Have any experience in providing PC to patients? n (%)					
Never	5 (8.06)	9 (3.26)	14 (4.14)	14.155	0.005^*^
Seldom	14 (22.58)	63 (22.83)	77 (22.78)		
Not sure	10 (16.13)	13 (4.71)	23 (6.8)		
Sometimes	18 (29.03)	83 (30.07)	101 (29.88)		
Often	15 (24.19)	108 (39.13)	123 (36.39)		

Remarks: * *Indicates P* < 0.05, and the difference is statistically significant

### Pharmaceutical care cognition and willingness to transform

The survey results demonstrated that, overall, nonclinical pharmacists’ understanding of PC was at an intermediate level. The judgement accuracy of the statement “PC providers are directly responsible for the patient’s health outcomes” was only 31.95%. The correct judgement rate of “PC is just a medication counselling service” was only 42.9%. The judgement accuracy of “PC is an extension of the current pharmacy services” was only 12.72%. Only 23.96% of respondents believed that “to provide PC, a consultation room or private area must be available.” The comparison of the constituent ratio of PC cognition between the willing and unwilling groups displayed no significant difference (*P* > 0.05), indicating that there was no significant difference in the constituent ratio of each item’s accuracy between the two groups (Table 3).

**Table 3:** Comparison of nonclinical pharmacists’ willingness to transform based on PC cognition

**Statement**	**Unwilling group(*n*** **= 62)**	**Willing group (*n* = 276)**	**Total (***n*** = 338)**	$\chi^2/\mathrm z$	***P***
**1. PC providers are directly responsible for the patient’s health outcomes, n (%)**					
Wrong answer	45 (72.58)	185 (67.03)	230 (68.05)	0.718	0.397
Correct answer	17 (27.42)	91 (32.97)	**108 (31.95)**		
**2. The primary aim of PC is to improve and maintain the patient’s quality of life, n (%)**					
Wrong answer	14 (22.58)	42 (15.22)	56 (16.57)	1.986	0.159
Correct answer	48 (77.42)	234 (84.78)	282 (83.43)		
**3. PC is just a medication counselling service [R], n (%)**					
Wrong answer	37 (59.68)	156 (56.52)	193 (57.10)	0.206	0.650
Correct answer	25 (40.32)	120 (43.48)	**145 (42.90)**		
**4. The term clinical pharmacy is interchangeable with PC [R], n (%)**					
Wrong answer	17 (27.42)	79 (28.62)	96 (28.40)	0.036	0.849
Correct answer	45 (72.58)	197 (71.38)	242 (71.60)		
**5. PC is an extension of the current pharmacy services [R], n (%)**					
Wrong answer	58 (93.55)	237 (85.87)	295 (87.28)	2.689	0.101
Correct answer	4 (6.45)	39 (14.13)	**43 (12.72)**		
**6. In PC, the pharmacist identifies and manages a patient’s existing and other potential drug therapy problems, n (%)**					
Wrong answer	6 (9.68)	21 (7.61)	27 (7.99)	0.081	0.777
Correct answer	56 (90.32)	255 (92.39)	311 (92.01)		
**7. PC involves a defined process of activities, all steps of which must be completed to provide this service, n (%)**					
Wrong answer	17 (27.42)	70 (25.36)	87 (25.74)	0.112	0.738
Correct answer	45 (72.58)	206 (74.64)	251(74.26)		
**8. All patients prescribed medicines require PC services, n (%)**					
Wrong answer	18 (29.03)	83 (30.07)	101 (29.88)	0.026	0.872
Correct answer	44 (70.97)	193 (69.93)	237 (70.12)		
**9. PC requires the availability of drug information resources, n (%)**					
Wrong answer	4 (6.45)	8 (2.90)	12 (3.55)	0.973	0.324
Correct answer	58 (93.55)	268 (97.10)	326 (96.45)		
**10. To provide PC, a consultation room or private area must be available [R], n (%)**					
Wrong answer	45 (72.58)	212 (76.81)	257 (76.04)	0.497	0.481
Correct answer	17 (27.42)	64 (23.19)	**81 (23.96)**		
**11. Provision of PC offers a feedback mechanism that optimizes the use of medicinal products, n (%)**					
Wrong answer	3 (4.84)	15 (5.43)	18 (5.33)	0.000	1.000
Correct answer	59 (95.16)	261 (94.57)	320 (94.67)		
**12. The patient’s active involvement is optional in the provision of PC[R], n (%)**					
Wrong answer	21 (33.87)	71 (25.72)	92 (27.22)	1.696	0.193
Correct answer	41 (66.13)	20 5(74.28)	246 (72.78)		
**Total score, M (P**_**25**_**, P**_**75**_**)**	6 (5,7)	6 (5,8)	6 (5,8)	0.89	0.625

### Pharmaceutical care attitude and willingness to transform

The scores of attitudes towards PC were compared between the willing and unwilling groups. The results demonstrated that there was a significant difference in the total score of the attitude scale between the two groups (*P* < 0.05) (Table 4).

**Table 4:** Comparison of nonclinical pharmacists’ willingness to transform based on PC attitudes

**Statement**	**Unwilling group (*n* = 62)**	**Willing group (*n* = 276)**	**合计(*n* = 338)**	***z***	***P***
Attitude scale score M (P25, P75)	35 (32, 39)	37 (34, 41)	37 (33, 41)	2.778	0.005*

Remarks: * *indicates P* < 0.05, and the difference is statistically significant

### Pharmaceutical care attitude and willingness to transform

Statistically significant factors, such as age, marriage, years of employment, professional title, PC experience, and PC attitude, were included in the logistic regression analysis. Quantitative data were converted into qualitative data based on the interquartile range, and the cut-off point was P25/P50/P75, divided into four segments.

The results of multivariate logistic regression analysis demonstrated that often providing PC to patients (*OR* = 4.601, 95% CI: 1.13–18.69, *P* < 0.05) and attitude towards PC (*OR* = 3.302, 95% CI: 1.19–9.19, *P* < 0.05) had statistical significance for the transformation intention of nonclinical pharmacists, as displayed in Table 5.

**Table 5:** Result of multifactor logistic regression

						**95% CI of OR**	
**Statement**	**B**	**SE**	**Wald**	***P***	**OR**	**Low**	**Up**
Do you have any experience in providing PC to patients? = 2	1.083	0.723	2.242	0.134	2.953	0.716	12.187
Do you have any experience in providing PC to patients? = 3	− 0.143	0.801	0.032	0.859	0.867	0.180	4.169
Do you have any experience in providing PC to patients? = 4	1.095	0.707	2.399	0.121	2.990	0.748	11.957
Do you have any experience in providing PC to patients? = 5	1.526	0.715	4.555	0.033^*^	4.601	1.133	18.689
Attitude scale score = 2	0.982	0.453	4.696	0.030^*^	2.670	1.098	6.488
Attitude scale score = 3	0.852	0.476	3.199	0.074	2.344	0.922	5.960
Attitude scale score = 4	1.194	0.522	5.230	0.022^*^	3.302	1.186	9.191

Remarks: * indicates *P* < 0.05, and the difference is statistically significant

## Discussion

With the change in the hospital pharmacy model, the Chinese government and nongovernmental and academic organizations are vigorously advocating clinical PC. A survey shows that most medical institutions in China have begun to provide all kinds of pharmaceutical services to patients, but this is still not perfect [13]. This study is the first large-scale survey of the willingness to transform nonclinical pharmacists in Xinjiang covering tertiary and secondary hospitals in various prefectures and cities. The samples are representative of the challenges that pharmacists face during the transformation process in Xinjiang. However, there are some limitations. First, according to the results of a literature review, the selection of possible influencing factors was not sufficiently comprehensive, and more possible factors should be included in future research. Second, there are information biases in the study. To limit information biases, the following efforts were made. At the stage of questionnaire design, objective indicators were included in the questionnaire as much as possible. Furthermore, we tried our best to design a strict and standardized quality control process to ensure the quality of the questionnaire. but information biases cannot be completely avoided. Among the 338 nonclinical pharmacists surveyed, 81.67% were willing to transform, showing that pharmacists who do not work in clinical pharmacies can be regarded as reserve teams that can quickly compensate for the lack of human resources of clinical pharmacists. However, approximately one-fifth of nonclinical pharmacists were unwilling to transform into clinical pharmacists. According to the survey results, the following problems were found.

### The attitude towards pharmaceutical care is not positive

According to the multivariate logistic regression analysis results, attitude towards PC is an important factor that affects the transformation of nonclinical pharmacists. The willingness to transform of pharmacists with an attitude score of 41 or more was 3.3 times higher than that of pharmacists who scored less than 32. The higher the attitude score, the more they recognized the provision of PC and the stronger their transformation intention was. The negative attitude of nonclinical pharmacists who are unwilling to transform indicates that changing attitudes is the key to promoting transformation.

### No understanding of clinical pharmacy and pharmaceutical care

Previous PC experience is an influential factor in the transformation of pharmacists. Pharmacists who frequently give patients PC are 4.6 times more eager to change careers than those who never give such care. This finding suggests that as pharmacists gain more knowledge of PC, they also come to recognize and embrace it, which increases their willingness to change careers to clinical pharmacists. Although the cognitive score of PC was not statistically significant in the difference in transformation intention, the median score of the overall score of the cognitive scale was six points, only reaching one-half of the total score. Nonclinical pharmacists have a general cognitive level of clinical pharmacy; however, most of them only understand macro concepts and have vague definitions of specific work content and responsibility subjects. For example, the judgement accuracy of the statement “PC providers are directly responsible for the patient’s health outcomes” was only 31.95%, while 57.1% of nonclinical pharmacists believed that “PC is just a medication counselling service.”

### High expectations for salary levels

The study's findings indicate that the majority of nonclinical pharmacists who are interested in changing careers believe that clinical pharmacists' salaries are higher. This also indicates that the performance income of nonclinical pharmacists has reached its limit or even declined as a result of the dramatic fall in the workload predominantly devoted to drug supply. Salary is an important factor in attracting pharmacists to become clinical pharmacists.

### Lack of motivation for transformation

This study found that the age and working years of pharmacists who were unwilling to transform were generally higher, and married pharmacists accounted for 83.87%, indicating that many older or married pharmacists are unwilling to leave their comfort zone [14]. On the other hand, their current positions lack challenge, their occupations are too steady, they are comfortable with the status quo, and they lack the incentive for self-improvement and transformation.

According to the above problems, the following suggestions are put forward.

### Strengthening the publicity and training of clinical pharmaceutical care

The health management department should be policy-oriented and strengthen the publicity of the development direction of the hospital pharmacy. Hospitals should take the publicity and specific responsibilities of PC as regular work and hold experience exchange meetings regularly. Clinical pharmacists should pay special attention to sharing their professional value experience and sense of acquisition to convey their professional and technical knowledge and their expertise in providing PC. Nonclinical pharmacists should be encouraged to gradually change their attitudes towards PC. The state should expand the recruitment of clinical pharmacists for post-training and require hospitals to include a certain number of pharmacists yearly and obtain clinical pharmacist qualification certificates to engage in clinical pharmaceutical work. In addition, hospitals should frequently hold popular science training courses related to the content and responsibilities of PC to deepen all pharmacists’ comprehensive understanding of this specialty, which is conducive to the transformation of the hospital pharmacy mode.

### Encourage nonclinical pharmacists to actively try primary PC

Most nonclinical pharmacists in hospitals are still engaged in drug-centred work, such as drug supply, dispensing, or preparation, so it is difficult for them to have the opportunity for direct contact with patients. Hospitals should encourage nonclinical pharmacists to enter the clinic, learn from clinical pharmacists how to help medical staff and patients solve drug use problems, and change passive service to active service [15]. They can begin with tasks such as prescription examinations and then advance to more complex activities such as pharmaceutical consultation, case discussion, and patient medication consultation. In doing so, they will obtain valuable expertise in clinical PC, raise the bar for pharmacist quality as a whole, and pave the way for a career change to clinical pharmacy.

### Improve the salary of clinical pharmacists

In China, whether in tertiary or grassroots hospitals, clinical pharmacists are not highly satisfied with their current work income and believe that their salary is lower than the value of their labour [16, 17]. Therefore, the Medical Security Bureau of Fujian Province of China issued the *Circular on the Trial Charging Policy of Pharmaceutical Care in Provincial Public Hospitals*, which was implemented on July 1, 2022. It lists 15 kinds of pharmaceutical service fees, ranging from 1 to 680 yuan, including drug treatment outpatient services, intravenous drug dispensing fees, drug sensitivity tests, and inpatient consultation fees. Among them, the outpatient fee for drug treatment is 20 yuan per time, which is included in the medical insurance payment scope according to regulations [18]. It is hoped that this policy can be successfully tested in Fujian Province and promoted nationwide to effectively improve the salary levels of clinical pharmacists. This will be a powerful measure to accelerate the transformation of pharmacists and promote the transformation of pharmaceutical models in hospitals.

### Reform of the performance appraisal system and the policy of professional titles

With the continuous progress of China’s medical reform, policies such as centralized drug procurement, DRG payment for a single disease, and performance evaluation of tertiary public hospitals are promoting the transformation of the pharmaceutical model. Change means challenges. The new position of a clinical pharmacist requires pharmacists to constantly learn new knowledge and meet higher requirements for their professional skills, which brings greater obstacles to married or senior pharmacists. However, there is no motivation without pressure. Relevant departments should comprehensively optimize the performance appraisal system of pharmaceuticals, formulate a new evaluation system according to the work scope of clinical pharmacists, and link it with pharmaceutical remuneration, postemployment, the promotion of professional titles, and other aspects to fully enhance the value of pharmacists and establish their position in rational drug use [19].

## Conclusions

In general, most nonclinical pharmacists in the hospital were willing to transition to clinical pharmacists, while others were hesitant due to their attitude and experience. Therefore, hospitals should strengthen publicity about clinical pharmacies, conduct training for pharmacists focusing on rational drug use, and encourage nonclinical pharmacists to gradually shift from “drug-centred” to “patient-centred.” It is also necessary to constantly improve the performance appraisal system of pharmaceutical management and to encourage pharmacists to leave their comfort zone, enhance their professional ability, and improve their work achievement and sense of self-worth to promote the transformation of the hospital PC mode.

## Data Availability

The datasets generated and/or analysed during the current study are not publicly available because the data used in this research are part of a larger dataset exclusively constructed with the authors’ efforts. This dataset is to be used in the authors’ other studies, which required confidentiality. However, they are available from the corresponding author on reasonable request.
